# Supplementary material for: Trends and projections of the global burden of T2DM-associated CKD related to high BMI: A global burden of disease study 2021
Source: PLoS One. 2025 Oct 10;20(10):e0333672. doi: 10.1371/journal.pone.0333672 (PMC12513629; doi:10.1371/journal.pone.0333672)
Supplement: Attachment — Submitted filename: Review Comments to the Author.docx [file pone.0333672.s003.docx]

Dear Academic Editor,

Please find below my consolidated reviewer comments for the manuscript entitled “Trends and Projections of the Global Burden of Type 2 Diabetic Nephropathy Related to High BMI: A Global Burden of Disease Study 2021”.

This research manuscript explores the global burden of type 2 diabetic nephropathy (T2DN) associated with high body mass index (BMI), using data from the Global Burden of Disease (GBD) Study 2021. The study analyzes historical trends from 1990 to 2021 and projects scenarios through 2050, examining mortality and disability-adjusted life years (DALYs). It highlights that high BMI is an important driver of increasing T2DN, with notable regional and sex disparities. The research emphasizes the urgency of interventions to prevent obesity and to improve access to healthcare.

I would recommend a Major Revision focused on clarifying the manuscript’s novel contribution, strengthening the methodological justification, and improving precision in terminology (chronic kidney disease due to type 2 diabetes) and the BMI cut points used. Implementing the suggested revisions would increase the manuscript’s clarity, reproducibility, and usefulness for policy-makers.

1. Context and novelty relative to recent studies

Why: Several recent GBD-based analyses address the burden of chronic kidney disease (CKD) attributable to type 2 diabetes and the role of high BMI (e.g., Wang et al., 2025 and others). Clarifying what is new in this manuscript will help readers and editors assess its added value.

Suggestion: The authors could add a brief paragraph in the Introduction that cites these recent studies and explains what the present manuscript adds (for example: projection horizon extended to 2050; an alternative projection method; more granular subregional/age/sex stratification; additional sensitivity analyses).

2. Clarify and justify the definition of “high BMI”

Why: Using thresholds such as 20–23 kg/m² labeled as “high BMI” can be confused with international categories for overweight/obesity (≥25/≥30 kg/m²). Interpreting results as “obesity-driven” without clarifying this distinction can be misleading.

Suggestion: The authors could clarify the exact source of these thresholds (e.g., GBD documentation, WHO Expert Consultation for Asian populations, or another reference), indicate which countries/regions apply which threshold (if applicable), and where appropriate use the term “elevated/high BMI” in Results and Conclusions instead of “obesity.”

3. Consistent terminology tied to the GBD definition (T2DM-Associated CKD / DKD)

Why: The term “diabetic nephropathy” usually implies clinical phenotypes (albuminuria, eGFR, KDIGO stages) that the GBD attributional category does not permit distinguishing. Using a neutral, explicit label avoids overinterpretation and is consistent with terminology adopted in other GBD-based works.

Suggestion: The authors could define and consistently use “T2DM-Associated CKD”, “CKD-T2DM” (or alternatively “Diabetic Kidney Disease, DKD” when used as a broad, non-phenotypic term). As noted in their Limitations (GBD does not distinguish proteinuria nor KDIGO stages), the Methods should state this explicitly.

4. Methodological details for reproducibility (Joinpoint, BAPC, code)

Why: Small modeling details influence replicability and the interpretation of projections.

Suggestion: The authors could provide methodological details in Methods: how data were imported into Joinpoint (R package or script), the maximum number of joinpoints allowed, the number of permutations used for permutation tests, significance thresholds, BAPC parameters (priors, MCMC iterations), software and package versions. If feasible, deposit analysis scripts in a public repository (e.g., GitHub, Zenodo) and provide the link.

5. Make the Data Availability Statement explicit

Why: Citing the GBD portal is helpful, but specifying the exact outputs improves reproducibility.

Suggestion: The authors could list the exact outputs extracted (deaths, DALYs, ASMR, ASDR), years, age groups, the cause definition used, and file formats. Providing the extracted dataset (CSV) as supplementary material would be useful.

Minor Revisions

GATHER checklist (recommended as supplementary material)

Why: Completing a GATHER checklist improves transparency for population-level estimates.

Suggestion: The authors could complete the GATHER checklist and include it as a supplementary file, indicating where each item is addressed in the manuscript.

Temper the discussion about under-ascertainment and propose validation

Why: Modeled GBD estimates may underestimate burden in regions with low registry coverage. Stating this explicitly and recommending validation improves interpretation.

Formatting and references: minor corrections

Why: Formatting and correct citations improve presentation and compliance with PLOS ONE style.

Suggestion: Correct references (Refs. 1, 10, 15, 23, 27). Add the comparative GBD references (including Wang et al., 2025) with DOIs and correct formatting. The authors could benefit from an English language copy-edit to improve clarity, grammar, and overall readability.

Sensitivity analysis and comparison with Wang et al. (2025) (recommended if feasible)

Why: Comparing projections and identifying concordances/divergences helps position the manuscript’s findings within the existing literature.

Suggestion: If feasible, include a sensitivity analysis using BMI ≥25 kg/m² and, in the Discussion, compare the present projections to 2050 with Wang et al.’s projections to 2036, discussing possible reasons for differences (definitions, methods, projection horizon).

References the authors should add and discuss in the Introduction and Discussion

1. Epidemiological status, development trends, and risk factors of disability-adjusted life years due to diabetic kidney disease: A systematic analysis of Global Burden of Disease Study 2021. doi:10.1097/CM9.0000000000003428.

2. Trends and analysis of risk factor differences in the global burden of chronic kidney disease due to type 2 diabetes from 1990 to 2021: A population-based study. doi:10.1097/CM9.0000000000003428.

3. Global burden and influencing factors of chronic kidney disease due to type 2 diabetes in adults aged 20–59 years, 1990–2019: a systematic analysis from the Global Burden of Disease Study 2019. https://doi.org/10.21203/rs.3.rs-3225375/v1.

4. Global, regional, and national burden of type 2 diabetes mellitus caused by high BMI from 1990 to 2021, and forecasts to 2045: analysis from the Global Burden of Disease Study 2021. (authors/title as provided by reviewer).

5. Y. Wang et al., 2025. Trends and analysis of risk factor differences in the global burden of chronic kidney disease due to type 2 diabetes from 1990 to 2021: A population-based study. Diabetes Obesity and Metabolism. https://doi.org/10.1111/dom.16183. Please cite and explicitly compare methods and projections (Wang et al. project to 2036) and state how the present analysis differs or adds value.

Addressing these points would significantly improve the manuscript’s methodological transparency, terminological precision, and compliance with PLOS ONE’s style and reporting expectations.

Thank you for the opportunity to review this work.
