# Supplementary material for: Trends and projections of the global burden of T2DM-associated CKD related to high BMI: A global burden of disease study 2021
Source: PLoS One. 2025 Oct 10;20(10):e0333672. doi: 10.1371/journal.pone.0333672 (PMC12513629; doi:10.1371/journal.pone.0333672)
Supplement: Attachment — Submitted filename: Response_to_Reviewers_auresp_2.docx [file pone.0333672.s005.docx]

Thank you for your thoughtful and constructive feedback on our manuscript. We greatly appreciate the time and effort you have put into reviewing our work. Below, we outline how we have addressed the points you raised:

Reviewer 1:

**Comment1**: *In the abstract/conclusions, highlight regional disparities and clarify that emerging therapies (GLP-1 RAs, SGLT2i) could alter future trajectories.*

**Response:** In response to your suggestion, we have incorporated a discussion on emerging therapies, including GLP-1 receptor agonists (RAs) and SGLT2 inhibitors (SGLT2i), in the abstract. We acknowledge the potential impact of these therapies on the future trends of T2DM-associated CKD, particularly in regions with a high disease burden. (page2, line 34-35)

**Comment2**: *Ensure consistent use of terminology (“high BMI” vs. “obesity”; harmonize with GBD definitions).*

**Response:** We truly appreciate your careful attention to the terminology used throughout the manuscript. In response to your comment regarding the consistent use of terminology (“high BMI” vs. “obesity”), we have carefully reviewed the manuscript and made the necessary revisions. Specifically, we have replaced instances of "obesity" with "overweight/obesity" to better align with the BMI thresholds defined in the methods section (BMI ≥ 25 kg/m²). This change ensures consistency and harmonizes the terminology with the Global Burden of Disease (GBD) definitions, as per your suggestion.

We believe this revision improves the clarity and precision of the manuscript. Thank you once again for your valuable input, and we hope the updated manuscript now meets your expectations.

**Comment3**: *Provide clarity on data availability (whether extracted CSV or repository link will be accessible).*

**Response:** We appreciate your suggestion to clarify the access to the data. As noted in the Methods section, we have provided the link to the relevant database. To further enhance the reproducibility of our work, we have also prepared the extracted data in CSV format as supplementary material. However, due to file size limitations, we were unable to upload the CSV file via the submission platform. If needed, we are happy to provide the data through alternative methods, such as via email or a file-sharing service, and will be glad to share it with you or the editorial team at your convenience. We hope this addresses your concern.

**Comment4**: *Simplify long sentences to improve readability, and standardize abbreviations in figures/tables.*

**Response:** Thank you for your valuable feedback. I have carefully considered your suggestions to simplify long sentences and improve readability. In response to your comment, I have revised several sections of the manuscript. The sentences in lines 59, 79-83, 160-164, 234-237, 257-260, 288-292, and 334-339 have been simplified for better clarity and flow. Additionally, I have standardized the abbreviations used in the figures and tables to ensure consistency throughout the manuscript. I believe these revisions significantly enhance the readability of the manuscript. Thank you again for your thoughtful suggestions, which have greatly contributed to improving the quality of the work.

**Comment5**: *Add one more policy example from Asia or Africa to balance global representation.*

**Response:** Thank you for your valuable feedback. Regarding your suggestion to include policy examples from Asia or Africa, we have incorporated the case of the Philippines—which implemented a sugar tax policy in 2018. The revised version adds the following statement: "In 2018, the Philippines implemented a 20% tax on sugary beverages, projected to prevent approximately 2,775 deaths over 20 years. This measure is expected to reduce 13,632 cases of ischemic heart disease and new cardiac events, 5,287 ischemic strokes, and 21,763 cases of T2D." (page17, line 328-331) This addition aims to present a more balanced perspective by incorporating policy examples from Southeast Asia and South Asia, providing broader context for tax measures addressing global health challenges stemming from unhealthy diets.

We sincerely thank the reviewers for their valuable suggestions on this paper. We have carefully revised each piece of feedback to enhance the accuracy and readability of the manuscript. We believe these modifications will significantly improve the quality of the article. Once again, we extend our gratitude for your valuable time and hard work.
